# A Record Review on the Health Status of Rohingya Refugees in Bangladesh

**DOI:** 10.7759/cureus.9753

**Published:** 2020-08-15

**Authors:** Taufique Joarder, Ipsita Sutradhar, Md Imran Hasan, Md. Mafizul I Bulbul

**Affiliations:** 1 Multisectoral Nutrition Project, FHI 360 Bangladesh, Dhaka, BGD; 2 Epidemiology and Public Health, BRAC James P Grant School of Public Health, BRAC University, Dhaka, BGD; 3 National Consultant, Institute of Public Health Nutrition, Dhaka, BGD; 4 Ministry of Health and Family Welfare, National Nutrition Services, Dhaka, BGD

**Keywords:** record review, health status, rohingya refugees, bangladesh

## Abstract

The Rohingya refugee crisis is neither new nor a sudden problem for Bangladesh. However, the recent violence in August 2017 instigated the migration of 6,93,000 additional Rohingyas into Bangladesh and as of June 2018, around one million Rohingya refugees were residing in Bangladesh. Against this backdrop, it is important to know their current health status because, without this information, equal and equitable health service provision is not possible. So, we conducted this review to understand the current health status of the Rohingya refugees in Bangladesh. For this purpose, a systematic literature search was conducted in July 2018 using transparent selection criteria and the keywords "Rohingya", "Health", Bangladesh". After screening the title and abstract and removing duplication, 12 articles and 21 organizational reports were found eligible for final review. Major health problems prevailing among Rohingya refugees are unexplained fever, acute respiratory infection, and diarrhea. Non-communicable diseases like hypertension, diabetes, and their risk factors are also highly prevalent among these people. More than half of the Rohingya refugees are women and many of them experience sexual abuse or exploitation. More than 50,000 Rohingya refugee women were pregnant, however, a significant portion of pregnant women did not have access to quality antenatal care. Mental health problems like post-traumatic stress disorder (PTSD), depression, and suicidal thoughts were also commonly prevailing in the Rohingya community.

## Introduction and background

The Rohingya refugee crisis is neither new nor a sudden problem for Bangladesh. After the introduction of the Emergency Immigration Act by the military regime of Myanmar in 1978, the minority Muslim Rohingyas started flocking to Bangladesh. The total number of Rohingya refugees in Bangladesh is debatable. The United Nations Refugee Agency (UNHCR) reported that in 2011, around 265,000 Rohingya Refugees were residing, out of which 29,000 were recognized, 36,000 were unrecognized, and 200,000 were undocumented by the Government of Bangladesh (GoB) and other non-governmental organizations (NGOs) [1].

However, the recent violence in August 2017 instigated the migration of 6,93,000 additional Rohingyas, increasing the number, as of June 2018, to around 1 million (918,936) in Bangladesh [2]. Refugees from the recent exodus found a place in large camps or settlements, and their total number is 904,056. Among these camps, the Kutupalong Expansion Site (an extension made near the original Kutupalong Refugee Camp) houses the majority of the refugees (610,251), followed by Hakimpara-Jamtoli-Bagghona Camp (98,529), and Nayapara Camp (71,562). The rest of the refugees are housed among the host communities of Cox’s Bazar Sadar, Ramu, Teknaf, and Ukhia (120,044).

The recent exodus of August 2017

Rohingyas live in the Rakhine state of Myanmar, adjacent to the Bangladesh border. The 1982 Citizenship Act of Myanmar created three unequal tiers in the citizenship of Myanmar, namely, Full Citizens (pink card), Associate Citizens (blue card), and Naturalized Citizens (green card), and thus disenfranchised several ethnic groups, including the Rohingyas [3]. As a result of alleged persecutions followed by the enaction of the law, a large number of Rohingyas eventually fled illegally to not only Bangladesh but also to countries such as Saudi Arabia, Pakistan, Thailand, and Malaysia [4]. Aung San Suu Kyi took up the role of the State Counselor of Myanmar in 2016. In Rakhine State, she restarted the citizenship verification process, which faced resistance from the Rohingya community [5]. The latest exodus of Rohingyas began on August 25, 2017, as the consequence of the Myanmar army's and Rakhine Buddhists’ campaign against Rohingya civilians followed by a coordinated attack of Arakan Rohingya Salvation Army (ARSA) on 30 police posts [5-6]. Within two months, from August 2017 to October 2017, hundreds of thousands of Rohingya refugees fled to Bangladesh through the Bangladesh-Myanmar border [2].

This massive influx made Rohingya refugees live in settlements where the majority of them did not have access to good housing, safe drinking water, and good sanitation systems, which, in turn, increased their vulnerability to a wide range of infectious diseases [2]. Additionally, Rohingya people are exposed to war-related traumatic events, including the destruction of property, loss of family members, witnessing extreme violence, and injury or loss of property. These events have the potential to make Rohingya refugees suffer from different psychological distress [5]. Against this backdrop, it is important to ensure health service for the Rohingya population, and to do so knowing about their current health status is imperative because without this information, equal and equitable health service provision, as well as appropriate resource allocation, is not possible. Besides, failure to provide adequate health service and thus to maintain the sound health of Rohingya refugees might adversely affect the health status of Bangladeshi people as well. So, we conducted this review to understand the current health status of the Rohingya refugees in Bangladesh. This will help the policymakers determine what evidence needs to be generated and how to better tackle the Rohingya situation in Bangladesh. This will also help international or bilateral agencies to prioritize their efforts for this protracted refugee crisis.

## Review

Methods

Search Strategy

A literature search was conducted in July 2018 using transparent selection criteria, to allow for a detailed analysis of the health issues of Rohingya refugees in Bangladesh. Both peer-reviewed and gray literature were searched separately by two researchers in databases: Google, Google Scholar, and PubMed. Keywords used for the literature search were: Rohingya, Health, and Bangladesh. As of July 2018, the number of articles found on Google Scholar using the keywords "Rohingya", "Health", Bangladesh" was 3,550 (616 since 2017). Using the same keywords on PubMed, we found 27 (using the keyword "Rohingya"); 4,06,7331 (using the keyword "Health"), and 17,730 (using the keyword "Bangladesh") articles. We also conducted manual searching to find out relevant organizational reports. We screened the titles and abstracts of all these articles and reports for initial inclusion. The following exclusion criteria were applied for further refinement and abridgment of the search.

Inclusion Criteria

§ Articles/organizational reports focused on the health issues of Rohingya people.

§ Articles/organizational reports based on both primary and secondary research.

§ Articles/organizational reports using both quantitative and qualitative approaches.

Exclusion Criteria

§ Articles/organizational reports published before the year 2017 (to get the most recent health status of Rohingya refugees reached with the recent exodus).

§ Articles/organizational reports on Rohingyas residing in Myanmar or countries other than Bangladesh (e.g., Thailand, Malaysia, Indonesia, India, Pakistan). However, multicounty studies that reported the health issues of Rohingyas residing in Bangladesh were included.

§ Correspondence, perspective, comments, letters, conference papers, and thesis/dissertation.

After screening the title and abstract and removing duplication, 12 articles were found eligible for review. Besides, 21 organizational reports from United Nations High Commission on Refugees (UNHCR), World Health Organization (WHO), Directorate General of Health Services (DGHS) of Bangladesh, Inter Sector Coordination Group (ISCG) on Rohingyas in Bangladesh, United Nations Children’s Fund (UNICEF), International Organization for Migration (IOM), Save the Children, United Nations Population Fund Bangladesh (UNFPA), and Medecin Sans Frontieres (MSF) published since 2017 were nominated for this paper, based on the above-mentioned inclusion and exclusion criteria.

Data Extraction

Two authors (TJ and IS) reviewed these documents carefully and extracted the findings in terms of the following headings:

§ Demographic information

§ Infectious diseases

§ Non-communicable diseases

§ Nutritional status

§ Child health

§ Mental health

§ Sexual and reproductive health

§ Gender-based violence

After data extraction, two authors (MMIB and MIH) crosschecked the tables to ensure consistency. Any disagreement that appeared during data extraction was resolved by group consensus. Finally, data analysis was performed using the thematic approach.

Results

Demographic Information

As of June 2018, nearly one million Rohingya refugees from 212,415 families are residing in Bangladesh, of which 904,056 reside in refugee camps and 120,044 reside in host communities [2]. Among newly-arrived Rohingya refugees, 54% were children, 60% were women and girls, and 10% were pregnant and lactating mothers [7].

Infectious Diseases

According to the Early Warning Alert and Response System (EWARS), the major health problems prevailing among Rohingya refugees are unexplained fever (2,27,928), acute respiratory infection (2,23,651), and diarrhea (1,92,560) [8]. Rohingya camps experienced a sudden outbreak of diphtheria in November 2017 and a measles outbreak in December 2017-April 2018 [8]. Though no system has been established yet to detect tuberculosis (TB) cases in Rohingya camps, it can be anticipated that TB cases are highly prevalent among Rohingya refugees considering the fact that Myanmar is one of the top 30 countries with the highest TB burden [9].

Non-Communicable Diseases

We hardly found any study estimating the prevalence of non-communicable diseases (NCDs) among Rohingya refugees in Bangladesh. BRAC conducted a need assessment in March 2018 and reported that 51.5% had hypertension and 14.2% had diabetes [10]. Besides, 36,930 persons were suffering from injuries [8]. NCD risk factors, such as smoking, using smokeless tobacco products, and indoor air pollution, are also highly prevalent among these people [11]. The actual burden of NCDs might currently be imperceptible due to the lack of efforts to detect these diseases in Rohingya camps [11].

Nutritional Deficiency

Nutritional deficiencies are highly prevalent among Rohingya refugees, especially among children. A recent population-based, cross-sectional study conducted in the Kutupalong refugee camp found that in children aged among six to 59 months, nearly half were suffering from stunting (height for age z-score <-2) and anemia and about one-fourth had Global Acute Malnutrition (GAM) (weight for height z-score: <-2 or bilateral pitting edema) [12]. Among Rohingya children, 4.1% and 4.2% were suffering from severe acute malnutrition (SAM) and moderate acute malnutrition (MAM), respectively [13]. The nutritional status of adolescent girls, as well as pregnant and lactating women in Rohingya camps, was also quite poor [13-14]. According to a survey conducted in 2018, around 10% of Rohingya women were undernourished [12].

Child Health

Evidence suggests that 54% of Rohingya refugees are children, and 703,000 of them need humanitarian assistance [7]. EWARS reported 82,382 consultations among under-five children through surveillance between the period August 25 and November 18, 2017 [8]. Among these, nearly one-third (32%) and just above one-fourth (27%) were cases of respiratory infections (ARIs) and unexplained fever, respectively. Cases of acute watery diarrhea, skin diseases, injuries, eye infections, and malaria were also found among this group of children [8]. Nutritional deficiencies like underweight, stunting, wasting, and iron deficiency anemia is highly prevalent among Rohingya children [12]. Female children also reported experiencing mental stress regarding their security issues, as they have to share toilets with males and they do not have private space in their tents for sleeping, bathing, and changing their clothes [15].

Sexual and Reproductive Health

Based on the latest UNHCR family counting exercise demographic data, more than half of the Rohingya refugees are women and around 316,000 of them are of reproductive age [13]. WHO reported that in February 2018, more than 50,000 women in the Rohingya community were pregnant [13]. Between February 2018 and May 2018, the expected cases of delivery and obstetrical complications were estimated at 16,513 and 2,477, respectively. The latest available data reveal that a significant portion of pregnant Rohingya women could not receive antenatal care (ANC) because of the unavailability or inaccessibility of the service [13,16]. In many sexual and reproductive health centers, there is a limited facility of essential ANC components such as blood testing, urine testing, and tetanus vaccination [16]. The utilization of health facilities is further hampered by the restriction in movements of the refugee population outside their respective camps [11,13].

Gender-Based Violence (GBV)

A recent study conducted in the Kutupalong and Nayapara refugee camps found that Rohingya women frequently experience sexual abuse or exploitation such as rape, forced sexual favors, and unwanted sex [17]. UNFPA, additionally, reported that more than 14,000 Rohingya girls and women experienced GBV between August 2017 and December 2017 [18]. Twelve point eight percent (12.8%) of women and girls experienced forced sexual favors, and 8.1% experienced forced and unwanted sex [17]. However, because of the stigma and humiliation associated with sexual violence, it is anticipated that GBV is underreported and the actual number of GBV victims are much higher [7].

Mental Health

There is a scarcity of evidence on mental health issues of Rohingya refugees residing in Bangladesh. A recently published cross-sectional study stated that 36.0% of Rohingya refugees were suffering from post-traumatic stress disorder (PTSD). Symptoms of depression and suicidal thoughts were also prevailing among 89.0% and 13.0% of people of the Rohingya community [17]. Female children also reported experiencing mental stress regarding their personal security, as they have to share toilets with the males and they do not have private space in their tents for sleeping, bathing, and changing their clothes [15]. Table 1 lists the current health status of Rohingya refugees in Bangladesh.

**Table 1:** Current health status of Rohingya refugees residing in Bangladesh (as of June 2018) N/A: Data were not available; Adult: Men and women of age 18 years or more; Children (U5): Under 5-year-old children; U18: Under 18-year-old girls; Women (RA): Reproductive age group women (15-49 years); * Values presented here represent the proportion of consultations (82,382) reported by EWARS between August 25, 2017, and November 18, 2017 HIV/AIDS: Human immunodeficiency virus infection and acquired immune deficiency syndrome; WHO: World Health Organization; EWARS: Early Warning Alert and Response System; SEAR: South-East Asia Region; UNFPA: United Nations Population Fund Bangladesh

Type of disease/symptom/health event	Name of disease/symptom/health event	Affected population	Total cases	Prevalence	Reference
Infectious Diseases	Unexplained fever	Adult, children	2,27,928	N/A	WHO, 2018a
	Acute respiratory infection	Adult, children	2,23,651	N/A	WHO, 2018a
	Diarrhea (watery and bloody)	Adult, children	1,92,560	N/A	WHO, 2018a
	Malaria	Adult, children	53	N/A	WHO, 2018a
	Measles/Rubella	Adult, children	1,410	N/A	EWARS, 2018
	Acute jaundice syndrome (Hepatitis A, B, C; Leptospira)	Adult, children	12,842	N/A	EWARS, 2018
	Measles (outbreak) (Dec 2017-Apr 2018)	Children (81% U5)	1,231		EWARS, 2018
	Diphtheria (outbreak)	Adult, children	7,772 (42 death)	N/A	EWARS, 2018
	Tuberculosis	Adult, children	4,000 (estimated)	N/A	WHO SEAR, 2018
	HIV/AIDS	Adult, children	5,000 (estimated)	N/A	WHO SEAR, 2018
Non-communicable Diseases	Hypertension	Adult	N/A	51.5%	Balsari et al., 2018
	Diabetes	Adult	N/A	14.2%	Balsari et al., 2018
	Injuries/Wounds	Adult, children	36,930	N/A	EWARS, 2018
Nutritional Deficiency	Stunting/chronic undernutrition	Children (U5)	N/A	43.4%	Leidman et al., 2018
	Global acute malnutrition (GAM)	Children (U5)	N/A	24.3%	Leidman et al., 2018
	Anemia	Children (U5)	N/A	47.9%	Leidman et al., 2018
		Women (RA)	N/A	57.2% (estimated)	WHO SEAR, 2018
	Severe acute malnutrition (SAM)	Children	7,796	4.1%	WHO, 2018a
	Moderate acute malnutrition (MAM)	Children	7,854	4.2%	WHO, 2018a
Child Health*	Acute respiratory infection	Children (U5)	N/A	32.0%	EWARS, 2018
	Unexplained fever	Children (U5)	N/A	27.0%	EWARS, 2018
	Acute watery diarrhea	Children (U5)	N/A	23.0%	EWARS, 2018
	Skin diseases	Children (U5)	N/A	6.0%	EWARS, 2018
Sexual and Reproductive Health	Pregnancy (Feb 2018)	Women (RA)	53,266	N/A	WHO, 2018c
	Expected delivery (Feb-May 2018)	Women (RA)	16,513	N/A	WHO, 2018c
	Obstetrical complications (Feb-May 2018)	Women (RA)	2,477 (estimated)	N/A	WHO, 2018c
Gender-Based Violence (GBV) Related	GBV (Aug 2017-Dec 2017)	Girls, women	>14,036	N/A	UNFPA, 2018
	Forced sexual favors	Girls, women	N/A	12.8%	Riley et al., 2017
	Forced and unwanted sex	Girls, women	N/A	8.1%	Riley et al., 2017
Mental Health	Post-traumatic stress disorder (PTSD)	Adult	N/A	36.0%	Riley et al., 2017
	Depressive symptoms	Adult	N/A	89.0%	Riley et al., 2017
	Suicidal thoughts	Adult	N/A	13.0%	Riley et al., 2017
	Feel afraid	Adult	N/A	14.0%	Riley et al., 2017

Discussion

Our review revealed that Rohingya camps are burdened with many infectious diseases such as respiratory tract infections and diarrhea. Infectious diseases are major contributors to ill health among refugee populations across the world [19-20]. A post-arrival medical assessment of European and African refugees in Western Australia also found that infectious diseases like TB (55.0%), hepatitis B (56.7%), syphilis (5.0%), malaria (8.0%), and giardiasis (9.5%) were quite common among the refugee population [21]. Infectious diseases alone or in combination with malnutrition are attributable to over three-quarters of deaths in conflict situations [22]. A study conducted among Rwandan refugees residing in the Democratic Republic of the Congo (DRC) reported that, immediately after arrival in DRC in 1994, 85% of total deaths were caused by diarrheal diseases (cholera, dysentery) [23]. The unavailability of safe drinking water, poor sanitation, overcrowded living place, and poor air quality in combination with poor immunity attributed to undernutrition makes the refugee population highly vulnerable to transmissible diseases [23-24]. If they cannot be controlled, these diseases, most alarmingly diphtheria, might be transmitted to the Bangladeshi host community and pose an adverse impact on the country’s health system. The provision of preventive, promotive, as well as curative, health services, therefore, needs to be ensured for the Rohingya population, to protect the health of the refugee community and host community.

It was found from our study that sexual and reproductive health is a significant concern among Rohingya refugees in Bangladesh. Previous research also found that refugees and forcedly displaced women are extremely vulnerable to negative sexual and reproductive health outcomes [25-26], mostly due to lack of knowledge on sexual and reproductive health issues such as menstruation, menopause, sexually transmitted diseases (STD), and cervical screening [25]. Having inadequate information on health service in a host country [27-28] and giving lower priority to one’s own health in comparison to concerns such as shelter, food, and other basic needs also play a role in this regard [29-30]. It is evident that women in humanitarian settings experience pregnancy-related complications and adverse birth outcomes more frequently when compared with pre-conflict situations [31-34]. Therefore, organizations working in Rohingya settlements should take the necessary steps to make reproductive health services available, accessible, and affordable; to ensure the quality of care and to increase sexual and reproductive health knowledge and awareness among refugee women. Using modern technologies, such as mobile phones, mobile apps, and social media, can be an approach to address these issues [35], however, some additional issues need to be considered carefully before using technology in Rohingya settlements. These include the availability of technologies among the target group, their level of education, and their health-related beliefs and norms [36].

Our study reveals the scarcity of evidence on NCDs among Rohingya refugees in Bangladesh. However, existing literature suggests that different non-communicable diseases like hypertension and diabetes are prevalent in Rohingya communities. This finding is in agreement with the finding of previous studies, in which NCDs were identified as major health concerns in humanitarian settings across the globe [37-43]. This finding might be explained by the high exposure of the refugee population to different behavioral and environmental risk factors of developing NCDs [44]. After migration, refugee populations often experience food insecurity and substantial dietary acculturation, which leads them to adopt unhealthy dietary habits [44-46]. Changes in family dynamics and responsibilities, along with the reduced scope of performing physical activity, make them lead a sedentary life [47-48]. Most importantly, mental stress appeared as a consequence of violence, and migration-related factors, such as adjusting to a different environment in a new country, intensify the vulnerability of refugee people to experience NCDs like hypertension [42,48].

## Conclusions

Based on our literature review, we strongly recommend context-specific strategies and a multistakeholder approach to address the health problems of the Rohingya refugees in Bangladesh. The GoB, in collaboration with national and international organizations, should generate and allocate financial resources to improve the availability, accessibility, and quality of health services provided to these people. Capacity building of service providers through the provision of systematic training on specific need-based issues can be beneficial. It is also imperative to place efficient and dedicated leaders in health facilities in that area. Further research is warranted to identify the health problems along with their distribution and determinants among Rohingya refugees. It is also essential to understand the knowledge, attitude, and perception of the Rohingya population regarding different health events, as well as their culture, beliefs, and norms of health-seeking behavior, to increase the utilization of health care service. Most importantly, all these activities to improve the health status of Rohingya refugees should comprehensively observe ethical issues. Besides, strides should be made to evaluate both the process and the effectiveness of the existing programs targeting the refugees. Finally, the GoB should continue cooperating with development and humanitarian partners until a conducive environment is created in Myanmar for them to return to their homes or a third-country settlement is agreed on.
